# Design principles for cost functions in optimal sensory coding

**DOI:** 10.3389/fncom.2026.1924396

**Published:** 2026-09-18

**Authors:** Veronika Koren, Stefano Panzeri

**Affiliations:** Institute for Neural Information Processing, University Medical Center Hamburg-Eppendorf (UKE), Hamburg, Germany

**Keywords:** correlations, efficient coding, neural coding, population coding, sensory coding, spiking neural networks

## Abstract

Studies of neural codes for sensory variables are rooted in the long-established principle that neural codes are organized to encode as much information as possible about sensory stimuli. This principle has been successful in explaining many features of neural activity, suggesting that a key component of a cost function determining sensory neural coding from first principles is the stimulus encoding error. However, recent work has shown that neural cost functions may have other terms reflecting costs or limitations of the biological hardware. One key piece is the metabolic cost of neural activity. Including metabolic cost in the cost function leads to optimized spiking models that exhibit spike-triggered adaptation. Another possible component of the neural cost function is reliable downstream transmission of information. Neurons have short integration time constants, which make it difficult for information to be transmitted reliably unless inputs to neurons are correlated. This suggests that cost functions accounting for constraints on downstream readout promote codes with redundancy-inducing correlations. We review and discuss evidence that neural codes in the brain have features consistent with the predictions expected if cost functions have additional terms beyond the encoding error. Adaptation currents are indeed present in biological neurons with the time scales predicted by metabolically efficient codes, and neural population codes have information-reducing correlations that support downstream information transmission. Finally, we discuss future directions important for fully establishing the different contributions to neural cost functions.

## Introduction

A central question for understanding biological intelligence concerns the principles determining how sensory information is encoded, processed and transmitted through the activity of neurons and neural populations. Seminal early work introduced the idea that sensory information processing should be analyzed in terms of the information carried by neural signals and the constraints imposed by their finite signaling capacity. Normative theories of neural computation proposed that sensory systems are organized to maximize information about external stimuli (Barlow, 1961; Laughlin, 1981), or to minimize errors in stimulus reconstruction, prediction, or dynamical representation (Olshausen and Field, 1996; Rao and Ballard, 1999). These theories have provided powerful frameworks for understanding neural activity in terms of the computations it supports. In particular, they have provided compelling explanations for observed neural activity features such as response non-linearities, adaptation to stimulus statistics, and redundancy reduction in early sensory systems (Simoncelli and Olshausen, 2001).

The optimality of a neural code depends on the criteria used to define it. These criteria are formalized by a cost function that needs to be minimized to achieve optimality. The traditionally considered cost function focuses on accurately encoding input stimuli and has been operationalized as the root mean squared error or minus the mutual information between the neurally-represented and target sensory signals. However, a cost function might contain other components in addition to the term for sensory encoding. Neural activity is metabolically expensive (Lennie, 2003), and spikes and synaptic transmission impose significant costs on the organism (Niven and Laughlin, 2008). Thus, a neural cost function may also contain a metabolic cost term that penalizes high activity levels. Adding metabolic constraints to the cost function can substantially change which sensory representations are optimal compared with costs based on encoding accuracy alone. Further, information present in one neural population is not necessarily fully available to downstream circuits. Downstream neurons read out information through biophysical mechanisms with short integration windows and non-linear input-output functions which are more effective when operating on correlated population activity. Therefore, cost functions may also include downstream-readout constraints and favor neural codes that differ from codes optimized only for stimulus encoding, such as codes with redundant correlations (Panzeri et al., 2022).

Here, we review studies suggesting that cost functions of biological neural populations may include constraints beyond those for optimal sensory coding. We focus on constraints that we investigated in our recent work, namely metabolic cost (Koren and Panzeri, 2022; Koren et al., 2025) and downstream readout (Runyan et al., 2017; Valente et al., 2021; Safaai et al., 2025; Ibáñez-Berganza et al., 2025), and discuss experimental observations supporting this view. We discuss both how these constraints can be applied to cost functions defined at the single neuron level, potentially explaining experimental properties of single neurons, or at the population level, potentially revealing the consequences for optimal computations of different types of relationships between neurons and enabling the design of optimal networks.

## Efficient coding based on sensory encoding and adaptation to stimulus statistics

An early formulation of efficient coding is the hypothesis that neurons and neural systems optimize a cost function maximizing the information they encode about external stimuli (Barlow, 1961; Attneave, 1954). A central result of studies investigating optimal neural coding of sensory information is that sensory coding must adapt to the statistical properties of the sensory stimuli. Because the neural response range is limited, efficient codes should allocate their range preferentially to stimulus values that occur frequently or carry high behavioral relevance, which means that optimal sensory responses should reflect key aspects of the statistics of the stimuli that an organism encounters.

Because of this, neurons and networks optimizing sensory coding must also adapt optimally to changes in stimulus statistics. Experimental and theoretical work has provided evidence that neurons in the sensory periphery and sensory cortex adjust their responses to changes in the distribution of sensory stimuli (Simoncelli and Olshausen, 2001). Fairhall et al. (2001) showed that a single neuron (H1) in the fly sensory system adapts to changes in stimulus variance by rescaling its input-output function. When stimulus variance increased, the input-output function became shallower and covered a wider range of stimulus values. When stimulus variance decreased, it instead became steeper and concentrated sensitivity over a narrower stimulus range. Mlynarski and Hermundstad (2021) extended this work considering the information-theoretic optimal adaptation of neural population responses to changes in stimulus statistics. They derived population codes that optimally trade-off the need to encode accurately the current incoming stimuli with the contrasting need to detect and adapt to sudden changes in stimulus statistics. These optimal population codes have properties closely resembling those observed in real cortical population responses during adaptation.

Together, these studies show the power of sensory-encoding-based cost functions to understand from first principles the responses of neurons and populations to sensory stimuli.

## Trade-off between encoding error and metabolic cost

Because neural activity is metabolically expensive, neural computations are likely constrained by metabolic expenditure (Attwell and Laughlin, 2001). Thus, several studies incorporated metabolic cost into single neuron or population cost functions. At the level of single neurons, Wang et al. (2016) analytically derived monotonic tuning curves that optimize information transmission for given stimulus statistics under metabolic constraints, finding that metabolic constraints strongly shape sensory tuning curves. When the metabolic constraint is not included, information-optimal tuning curves follow the histogram equalization principle suggested by Laughlin (1981), so that neural response levels are used uniformly across the available dynamic range. Including the metabolic constraint instead biases responses toward lower firing rates.

Other models introduced metabolic constraints at the level of neural populations. Instead of focusing on how neural populations minimize their encoding error when stimulus statistics changes, these studies asked how a neural population optimizes its parameters for a fixed stimulus distribution under both sensory coding and metabolic constraints. The problem of designing such efficient recurrent networks has been addressed first with rate models (Zhu and Rozell, 2013; Kafashan and Ching, 2017; Rullán Buxó and Pillow, 2020; Prat-Carrabin et al., 2026; Kadmon et al., 2020) and then with spiking-neuron models (hereafter termed models of efficient coding with spikes). Here, we focus on models of efficient coding with spikes because they are more biologically plausible.

These models have considered how a whole recurrent neural network can minimizes the sum of a population-level encoding error of a time-dependent stimulus signal and a population-level metabolic cost of spiking (Denève and Machens, 2016). The encoding error penalizes inaccurate representations of the target signal, whereas the metabolic cost penalizes recent spiking activity. Minimizing this efficiency cost function therefore favors accurate signal representation with few spikes. The occurrence of each spike is conditioned on a decrease of the instantaneous loss. Each spike is thus timed to decrease the loss. More generally, fine-grained spike timing allows more information to be processed with the same number of spikes and may reduce energy cost relative to implementations not exploiting it (Verzi et al., 2018).

The efficient-coding cost function can be implemented by recurrently connected networks of leaky integrate-and-fire neurons (Bourdoukan et al., 2012). The structured recurrent connectivity derived from the cost function shapes network activity in both stimulus-driven and spontaneous states (Koren and Denève, 2017). Efficient spiking models reproduce empirically observed levels of single-neuron variability and yield neural representations that are robust to neuronal loss (Barrett et al., 2016), neuron-intrinsic noise, synaptic delays (Chalk et al., 2018, 2016) and perturbations to population activity (Calaim et al., 2022). Moreover, local learning rules can approximate the optimal synaptic connectivity prescribed by analytical derivations of efficiency cost functions by maintaining time-dependent balance between excitatory (E) and inhibitory (I) synaptic currents in the presence of error feedback signal (Alemi et al., 2018; Denève et al., 2017).

A key property of biological networks is Dale's law: a neuron uses the same neurotransmitter on all of its outgoing synapses. Unlike what happens when using cost functions that do not separate neurons into cell types, assigning separate cost functions to E and I neurons yields E-I efficient spiking networks that obey Dale's law (Koren and Panzeri, 2022; Boerlin et al., 2013). By design, efficient E-I spiking networks keep firing rates and representational error bounded at all times (Urdu et al., 2025). In such E-I efficient networks, parameters that optimize the trade-off between average encoding error and metabolic cost correspond to a biologically realistic parameter set that matches measurements from primary sensory cortex (Koren et al., 2025). These results indicate that E-I-specific cost functions that trade off encoding accuracy against metabolic cost can capture key properties of sensory cortical networks and highlight the relevance of cell-type-specific cost functions.

In a series of studies, we and others investigated the effect of including metabolic cost in the cost function in networks designed to encode low-dimensional time-varying signals, that is, signals made of few independent features (Koren and Panzeri, 2022; Koren et al., 2025; Koren and Denève, 2017; Boerlin et al., 2013; Gutierrez and Denève, 2019; Zeldenrust et al., 2021). In such networks, metabolic cost adds a constant term to the firing threshold (Boerlin et al., 2013), reducing spike probability. When single-neuron and population readouts have different time constants, the same cost function also prescribes neuron-intrinsic currents (Koren and Panzeri, 2022). Under additional constraints, these currents can produce slow (Gutierrez and Denève, 2019) or fast spike-triggered adaptation (Koren et al., 2025). Spike-triggered adaptation transiently hyperpolarizes the membrane potential after a spike and reduces subsequent excitability, with an amplitude that increases with the relative weight of metabolic cost in the cost function. Thus, metabolic cost does not only suppress firing but also changes the timing of spikes selected by the efficiency cost function. These results suggest that the after hyperpolarization currents widely observed in biological neurons (Shao et al., 1999; Storm, 1987) may be understood as the result of optimizing sensory coding while controlling its metabolic cost.

We asked whether adding metabolic cost to sensory coding cost affects the optimized network parameters and resulting neural response features (Koren and Panzeri, 2022; Koren et al., 2025). We first derived properties of networks designed to optimize a cost function that includes both encoding error and metabolic cost. To illustrate the specific contribution of the metabolic cost, we then compared networks optimized either for encoding error alone or for the sum of encoding error and metabolic cost, while keeping everything else identical (Figure 1A). We jointly optimized four parameters: the membrane time constant, the noise intensity, the ratio of E to I neuron numbers, and the ratio of average I-I to E-I connectivity strength. After optimization, both models tracked time-dependent target signals with similar accuracy (Figure 1B, black for target signals and red for E-population estimates). However, they converged to distinct parameter regimes. Compared with networks optimizing encoding error and metabolic cost, networks optimizing encoding error alone had lower noise and weaker inhibition, while the E-I neuron number ratio did not differ systematically between the two cost functions (Figure 1C, black and magenta for networks optimizing error alone and the sum of error and cost). Differences in optimized parameters translated into differences in network activity (Figure 1D). The network optimizing the encoding error alone had slightly higher firing rates and significantly lower trial-to-trial variability. In particular, the network including metabolic cost had Fano Factor (the ratio of the variance to the mean across trials of spike counts) close to 1, whereas the network optimizing only the coding error had Fano Factor close to 0.5. These differences are consistent with the lower noise intensity and weaker inhibition (Figure 1C). Thus, optimizing metabolic cost does not simply reduce firing, but shapes response variability. Because the trial-to-trial variability of sensory cortical neurons is close to that of a Poisson process (Churchland et al., 2010), including metabolic cost makes the activity of efficient spiking networks more biologically realistic.

**Figure 1:**
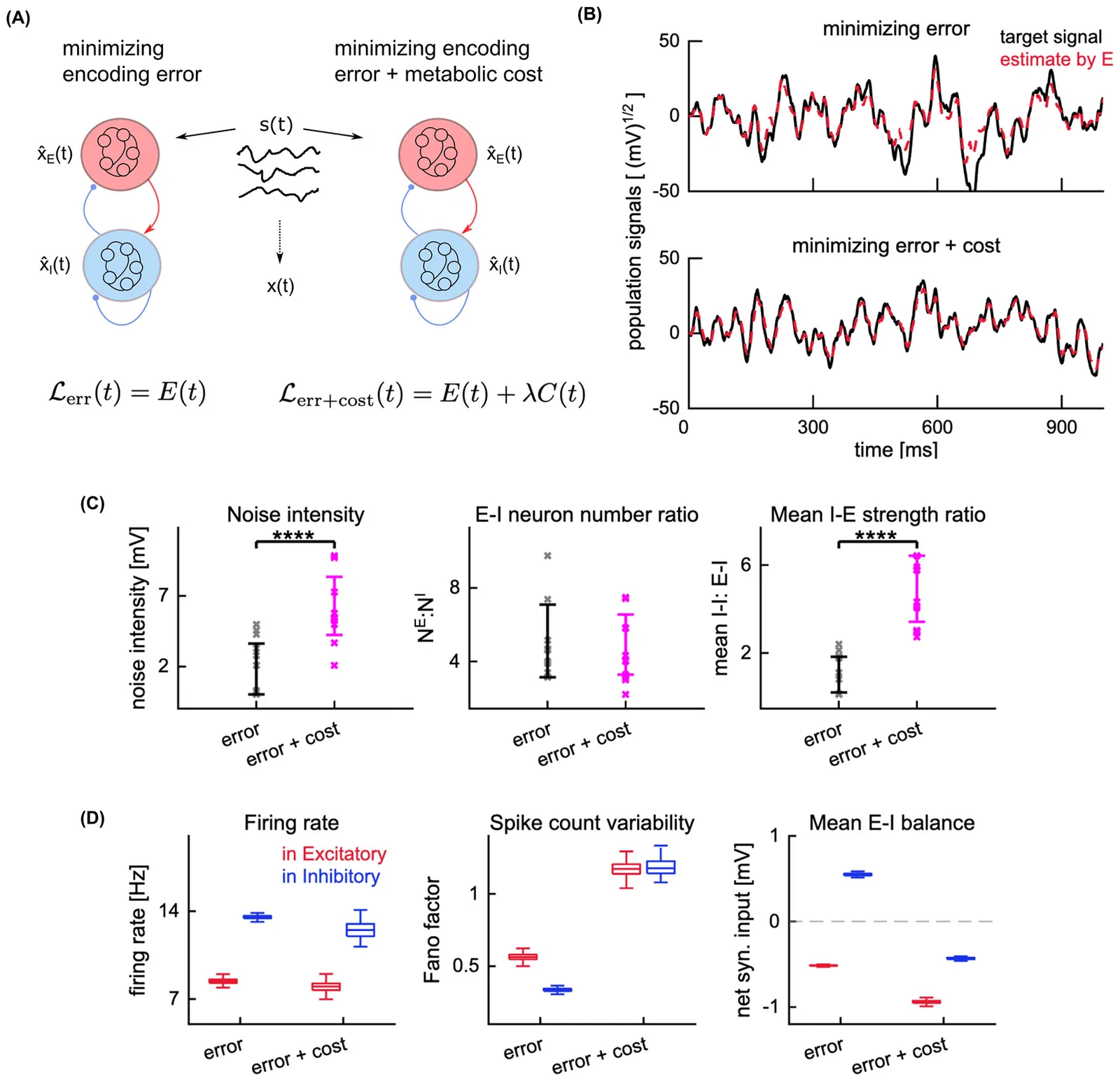
Metabolic cost changes networks optimized for encoding accuracy. **(A)** Schematic of the optimization procedure. We optimized networks minimizing either the encoding error alone **(left)** or a linear sum of encoding error and metabolic cost (right; as in Ref. Koren et al., 2025). Both models have the same E-I architecture, receive the same stimulus s(t), and estimate the same target signal x(t). The encoding error is the average of the E- and I-population encoding errors. The E-population encoding error is the squared error between the E-population readout and the target signal x(t). The I-population encoding error is the squared error between the E- and I-population readouts. The stimulus consisted of three independent Ornstein-Uhlenbeck processes. **(B)** The target signal (solid black) and E-population readout (signal estimate; dashed red) in the best performing network minimizing the encoding error alone **(top)** or the sum of the encoding error and the metabolic cost **(bottom)**. The best network minimizing the linear sum of encoding error and metabolic cost recovered the same set of parameters as reported in Ref. (Koren et al., 2025). **(C)** Parameters of the 15 best networks (lowest training loss) minimizing the encoding error alone (black) or the sum of the encoding error and the metabolic cost (magenta). The error bars show the mean ± standard deviation. The non-zero optimal noise levels found in both network types **(left)** are consistent with analytical results (Timcheck et al., 2022) **(D)** Firing rate **(left)**, Fano factor **(middle)** and the time-averaged net sum of E and I synaptic inputs to single neurons **(right)** computed on the best performing network of each type. Results are shown for E (red) and I neurons (blue). I neurons have higher firing rates and therefore higher metabolic expenditure than E neurons in both network types. The Fano factor, which measures spike-count variability, is significantly lower in the network minimizing the encoding error alone. Further, the net synaptic input in I neurons is dominated by excitation in the network minimizing the encoding error and dominated by inhibition in the network minimizing the sum of the encoding error and the metabolic cost, indicating a transition between the two network types from an excitation-dominated to an inhibition-dominated regime in I neurons. In both network types, the time-averaged net synaptic input is also more negative in E than in I neurons. Each distribution is across neurons. For (**B–D)**, we used 100 parameter initializations and 5 random seeds. All initializations reached convergence. For (**B)** and **(D)**, we tested network performance on new Ornstein-Uhlenbeck stimuli generated with the same parameters as the training stimuli. For (**D)**, we used 100 simulation trials of 1 s duration and averaged results across trials. The Fano factor was measured on spike counts in 100 ms time bins. Please note that panels C (middle), C (right), and D (middle) report unitless quantities.

Further, a cost function with only encoding-error optimization prescribes structured recurrent connectivity that in turn promotes temporal E-I balance, defined as the ability to maintain a tight balance of E and I synaptic inputs to each neuron at each time instant (Denève and Machens, 2016). Including the metabolic constraint increased temporal E-I balance (Koren et al., 2025) and enforced an inhibition-dominated regime, in which I synaptic inputs were stronger than E inputs (Figure 1D, right; see ref Koren et al., 2025). Including the metabolic cost therefore resulted in neural dynamics consistent with two empirical observations in sensory cortex: synaptic excitation and inhibition are coordinated over time (Okun and Lampl, 2008), and inhibition often dominates (Haider et al., 2013).

## Trade-offs between information encoding and information transmission

The notion that neural codes optimize information encoding has also permeated the study of neural population codes (Dupret et al., 2026; Nigam et al., 2019; Averbeck et al., 2006). Many studies considered which features of population activity enhance the amount of information encoded in a neural population, under the assumption that neural population codes maximize encoding of information within the same population. For example, several studies have investigated how the information encoded by populations depends on the sparseness of neural representations (intuitively, the fraction of active neurons at any given time), finding that very low values of sparseness maximize the information per spike, a measure which combines encoding quality and metabolic cost (Dupret et al., 2026; Finkelstein et al., 2018; Kira et al., 2023).

A feature of neural activity that has received substantial attention in terms of its contribution to the encoded information is the correlations between neurons (Panzeri et al., 2022; Averbeck et al., 2006). Modeling studies have investigated how the strength and structure of correlations between neurons affect the amount of information about sensory stimuli that the responses of a population carry (Abbott and Dayan, 1999; Panzeri et al., 1999; Averbeck and Lee, 2006). Correlations between the activity of different neurons can either increase, decrease or leave unchanged the information carried by a population. Their effect depends on several factors, the main one being the relative sign of noise and signal correlations. Noise correlations quantify the correlations between the activity of neurons at fixed stimulus. Signal correlations quantify the correlations between the trial-averaged activity across different stimuli. When signal and noise correlations have the same sign, noise correlations decrease information (Figure 2A, left). When they have opposite signs, noise correlations increase information (Figure 2A, right). In most experimental studies on sensory cortices or decision-making areas, noise correlations were found to decrease information. This was shown by comparing information decoded from the real simultaneously recorded responses of the neural population (which contains noise correlations) and the decoding of the stimulus from pseudo-population responses obtained from randomly shuffling responses of different neurons from trial to the same stimulus to destroy noise correlations (as in Figure 2A, center). The information in shuffled responses is often higher than that in the real responses (Panzeri et al., 2022; Bartolo et al., 2020; Rumyantsev et al., 2020; Golledge et al., 2003).

**Figure 2:**
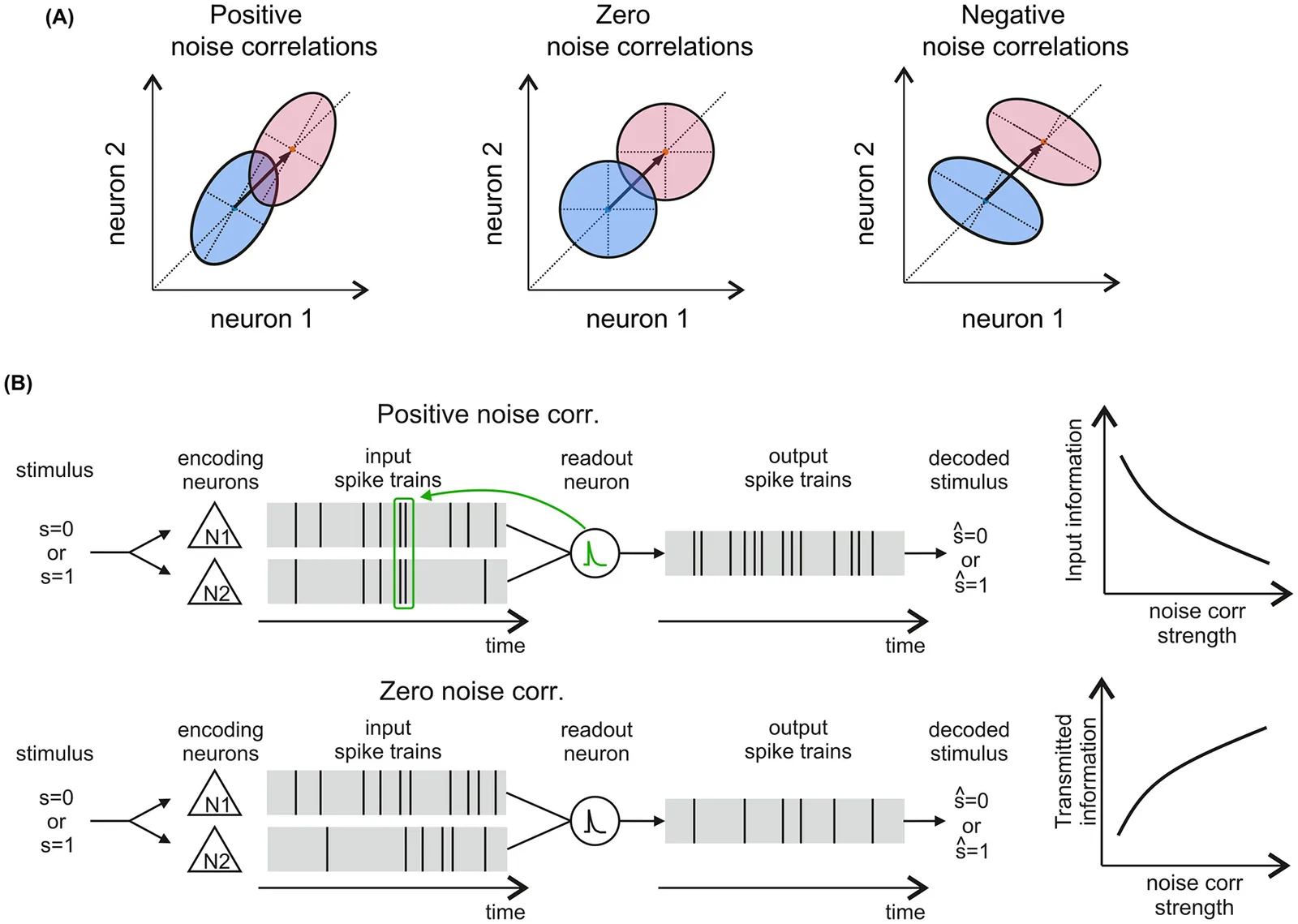
Illustration of how noise correlations shape information encoding and transmission. **(A)** We use cartoons of response distributions (ellipses) of populations of neurons (*N*_1_, *N*_2_) in the space of neural population activity (using *n* = 2 neurons) in response to two stimuli (blue and red). Across panels, single neuron properties are the same but noise correlations vary. Left: Positive noise correlations between neurons with positive signal correlations decrease the amount of encoded information about stimulus (overlapping distributions being harder to discriminate) with respect to population activity without noise correlations (as on the Middle panel, in which noise correlations are absent). Noise correlations with sign opposite to those of signal correlations (e.g., the former negative and the latter positive, as in the Right panel) increase stimulus information. Stimulus information in these figures can be conceptualized as the inverse of the overlap of the two distributions: the less overlap there is, the fewer decoding errors there will be. **(B)** Transmission of information through coincidence detection. Left panels: A neuron with a short integration time constant (green window) that receives stimulus-modulated presynaptic input spike trains from two different neurons produces a stronger output firing rate when receiving correlated (top left) than uncorrelated **(bottom left)** inputs. This is because correlations increase the number of time points with coincident input spikes compared with when receiving inputs with the same individual-neuron characteristics but no correlations. Right panels: analysis of the models shows that although input correlations reduce the stimulus information encoded in the inputs, the stimulus information transmitted by the output neuron can increase with the strength of input correlations.

These results suggest that a neural code optimized solely for population-level encoding should not contain information-limiting correlations. However, such correlations are commonly found in cortical data (Panzeri et al., 2022; Averbeck et al., 2006). Does this imply that neural population codes are not designed optimally? Or does it mean that they are designed optimally with a cost function containing terms other than encoding? A clue to this answer was found in a series of recordings from mouse cortex during perceptual decisions, which reported that correlations were information limiting but yet were stronger in trials in which mice made correct perceptual judgments, compared to trials when mice made incorrect judgements (Runyan et al., 2017; Valente et al., 2021; Safaai et al., 2025; Francis et al., 2022).

One possibility to explain these results is that the downstream readout of information to produce choices is facilitated by correlations. We demonstrated this in a recent study in which we constructed a statistical model of single-trial choices made by the mouse based on the information in neural activity in the same trial. We found that the probability of making a choice that followed the stimulus inferred as the most likely by decoding neural activity was higher when responses of neurons were more correlated (Valente et al., 2021; Safaai et al., 2025; Francis et al., 2022). This implies that information encoded in the activity of neurons can be more effectively used for downstream decisions if responses have some level of correlations. We also studied this with biophysical models of propagation of synaptic signals through the membrane of a neuron (Figure 2B). Because of short membrane integration time constants, activity is better propagated if correlations cluster spikes closer to each other in time (Figure 2B). An analytical study of this model (Ibáñez-Berganza et al., 2025) shows that moderate, intermediate correlation strength maximizes the amount of information transmitted through the membrane of the neuron (Figure 2B). The values of correlations maximizing the amount of transmitted information are roughly comparable to the range of values observed in cortical neurons (Ibáñez-Berganza et al., 2025).

These results suggests that neural codes are not optimized to encode information locally, but to trade off the need to encode information with the need to transmit it. How to optimally trade off these demands may depend on the primary function of the brain area, with a study suggesting that population codes in sensory areas have weaker correlations to weigh more sensory coding and populations in decision-making areas have stronger correlations to weigh more downstream transmission (Runyan et al., 2017).

## Discussion

Sensory-encoding-based neural cost functions have proven successful for explaining response properties of sensory neurons. We reviewed how adding additional biologically-plausible constrains may change these results. Adding constraints on metabolic expenditure leads to designing biologically plausible E-I networks that reproduce additional single-neuron and network-level properties of sensory cortex. Adding constraints on downstream readability leads to population codes that allow redundant correlations to promote information transmission to downstream areas at the cost of possibly reducing the encoded information.

A first direction for future work is to understand how to jointly optimize encoding accuracy, transmission to downstream readouts, and metabolic cost. Multilayer spiking networks provide a natural setting, because they allow cost functions that constrain both the encoding of information at one layer and their transmission across layers. Such models could form important theoretical counterparts to empirical studies of organization of neural codes in projection pathways (Safaai et al., 2025). We hypothesize that incorporating information transmission into cost functions would lead to networks with higher across-neuron correlation strength.

A second direction is to ask how richer computations can be implemented efficiently. Cortical neurons often non-linearly combine different afferent features. For example, primary somatosensory neurons non-linearly combine information from different types of skin receptors (Emanuel et al., 2021). It remains unknown how to capture complex feature interactions, for example mutually amplificatory and suppressive interactions, in efficient coding models. Other important computations include linear dynamical signal transformations, such as converting transient inputs into damped oscillations (Bourdoukan and Denève, 2015), and non-linear transformations, such as generating persistent attractor states (Alemi et al., 2018).

A third direction is to understand the relationship between efficient coding and low-rank recurrent networks (Mastrogiuseppe and Ostojic, 2018; Cimeša et al., 2023), especially those producing target outputs that solve cognitive tasks (Dubreuil et al., 2022). The recurrent connectivity optimizing cost functions with sensory and metabolic cost has low-rank structure determined by the dimensionality of the encoded stimuli (Koren et al., 2025; Boerlin et al., 2013). It would be interesting to examine how metabolic cost may change the optimal connectivity rank, for example selecting among degenerate solutions by preserving stiff dimensions that strongly affect coding performance while suppressing energetically costly dimensions with little coding benefit.
